# Cigarette tar accelerates atherosclerosis progression via RIPK3-dependent necroptosis mediated by endoplasmic reticulum stress in vascular smooth muscle cells

**DOI:** 10.1186/s12964-024-01480-6

**Published:** 2024-01-16

**Authors:** Xiaoxuan Bai, Ying Wang, Xing Luo, Xiaoyi Bao, Xiuzhu Weng, Yuwu Chen, Shan Zhang, Ying Lv, Xinyu Dai, Ming Zeng, Dan Yang, Sining Hu, Ji Li, Yong Ji, Haibo Jia, Bo Yu

**Affiliations:** 1 Department of Cardiology, The Second Affiliated Hospital of Harbin Medical University, Harbin, 150001 China; 2 National Key Laboratory of Frigid Zone Cardiovascular Diseases, Harbin Medical University, Harbin, 150001 China; 3 Key Laboratory of Myocardial Ischemia, Ministry of Education, Harbin Medical University, Harbin, 150001 China; 4 Department of Forensic Medicine, Harbin Medical University, Harbin, 150081 China; 5 Department of Pharmacology (State-Province Key Laboratories of Biomedicine-Pharmaceutics of China), Key Laboratory of Cardiovascular Medicine Research and NHC Key Laboratory of Cell Transplantation, Harbin, 150001 China

**Keywords:** Cigarette tar, Atherosclerosis, Vascular smooth muscle cell, Necroptosis, Endoplasmic reticulum stress

## Abstract

**Background:**

Tar is the main toxic of cigarettes, and its effect on atherosclerosis progression and the underlying mechanisms remain largely unknown. Vascular smooth muscle cells (VSMCs) play a key role in atherogenesis and plaque vulnerability. The present study sought to investigate the mechanism of atherosclerosis progression through tar-induced VSMC necroptosis, a recently described form of necrosis.

**Methods:**

The effect of tar on atherosclerosis progression and VSMC necroptosis was examined in ApoE^−/−^ mice and cultured VSMCs. The role of necroptosis in tar-induced plaque development was evaluated in RIPK3-deletion mice (ApoE^−/−^RIPK3^−/−^). The key proteins of necroptosis in carotid plaques of smokers and non-smokers were also examined. Quantitative proteomics of mice aortas was conducted to further investigate the underlying mechanism. Pharmacological approaches were then applied to modulate the expression of targets to verify the regulatory process of tar-induced necroptosis.

**Results:**

Tar administration led to increased atherosclerotic plaque area and reduced collagen and VSMCs in ApoE^−/−^ mice. The expression of RIPK1、RIPK3、and MLKL in VSMCs of plaques were all increased in tar-exposed mice and smokers. RIPK3 deletion protected against VSMC loss and plaque progression stimulated by tar. In mechanistic studies, quantitative proteomics analysis of ApoE^−/−^ mice aortas suggested that tar triggered endoplasmic reticulum (ER) stress. PERK-eIF2α-CHOP axis was activated in tar-treated VSMCs and atherosclerotic plaque. Inhibition of ER stress using 4PBA significantly reduced plaque progression and VSMC necroptosis. Further study revealed that ER stress resulted in calcium (Ca^2+^) release into mitochondria and cytoplasm. Elevated Ca^2+^ levels lead to mitochondrial dysfunction and excessive reactive oxygen species (ROS) production, which consequently promote RIPK3-dependent necroptosis. In addition, Ca^2+^/calmodulin-dependent protein kinase II (CaMKII) activated by cytosolic Ca^2+^ overload binds to RIPK3, accounting for necroptosis.

**Conclusion:**

The findings revealed that cigarette tar promoted atherosclerosis progression by inducing RIPK3-dependent VSMC necroptosis and identified novel avenues of ER stress and Ca^2+^ overload.

**Supplementary Information:**

The online version contains supplementary material available at 10.1186/s12964-024-01480-6.

## Introduction

Cigarette smoking is a widely recognized major risk factor for atherosclerotic disease [1]. Even after quitting smoking, the increased risk of coronary heart disease can persist for up to 20 years [2]. A large body of evidence has indicated that smoking accelerates atherosclerosis and vulnerable plaque formation [3–5]. Over the past few decades, efforts have been made to reduce the harmful effects of smoking by lowering tar yield, the primary toxic component in cigarettes [6]. However, few studies have explored the direct impact of tar on atherosclerosis progression. Certain polycyclic aromatic hydrocarbons in tar have been found to activate phospholipase A2 in human coronary artery endothelial cells, leading to apoptosis as well as inducing a proinflammatory state in macrophages [7–9]. Despite the reported association of certain components of tar with atherosclerosis, the underlying mechanisms of the pro-atherosclerotic effects of tar remain unclear.

VSMCs play a crucial role in atherosclerosis progression [10, 11], as they are the sole cells capable of producing collagen within plaques. Loss of VSMCs via initiation cell death leads to the thinning of the fibrous cap and the promotion of necrotic core formation [12]. While VSMC death was previously thought to be non-programmed necrosis, research has shown that VSMCs can undergo various programmed cell death pathways [13, 14]. However, the specific impact of tar on VSMCs and its mechanism in the progression of atherosclerosis remains uncertain.

Necroptosis is a distinct type of programmed cell death characterized by cellular organelle swelling and cell membrane rupture [15]. Upon induction of classical necroptosis, receptor-interacting protein kinase 1 (RIPK1) binds with receptor-interacting protein kinase 3 (RIPK3) to form an amyloidal death complex that activates their kinase activities. Activated RIPK3 recruits and phosphorylates a downstream effector protein mixed lineage kinase domain-like protein (MLKL), which is specifically required for necroptosis execution [16]. Then, abundant damage-associated molecular patterns (DAMPs) are released, contributing to a persistent inflammatory response. Studies have shown increased levels of necroptosis-related mRNA and protein in the lung tissue of cigarette-exposed mice [17]. However, whether necroptosis contributes to the effect of tar on atherosclerosis progression remains largely unclear.

ER is a vital organelle involved in protein secretion, folding, translocation, Ca^2+^ homeostasis, and lipid and steroid biosynthesis [18]. Internal and external stressors, such as starvation, hypoxia, and toxic substances, can stimulate the ER, leading to unfolded or misfolded proteins to accumulate in the lumen, and a pathological state of ER dysfunction is created, called ER stress [19]. Prolonged ER stress can cause cell death and activate inflammatory response pathways. While it is known that ER stress is linked to atherosclerosis, the role of tar stimulation in inducing ER stress and its impact on VSMC death remains unclear [20].

In this study, by employing ApoE^−/−^ mice and specific genetic deletion mice (RIPK3^−/−^), we aimed to determine the effect and molecular mechanisms of tar on VSMCs and atherosclerosis progression. Moreover, a quantitative proteomics analysis of mice aortas was conducted to explore the potential mechanism by which tar induces VSMC necroptosis. Finally, we examined the role of Ca^2+^ overload in the regulation process of tar-induced necroptosis.

## Materials and methods

### Human atherosclerotic lesion analysis and ethics

Human carotid atherosclerotic plaques were obtained from patients undergoing surgery for carotid endarterectomy in Xuanwu Hospital of Capital Medical University (Ethical code: SYDW2019–259). All samples were collected with informed consent and procedures were performed according to the Declaration of Helsinki. Tissues were immediately preserved at -80 °C after removal and divided into two groups according to smoking or not.

### Animals

All animal study protocols were approved by the Animal Care and Use Committee of the Second Affiliated Hospital of Harbin Medical University (Approval No. YJSDW2022–244) and followed the recommendations of the European Ethical Committee (EEC) (2010/63/EU). ApoE^−/−^ and RIPK3^−/−^ mice on a C57BL/6 N background were purchased from Cyagen Biosciences (China) and housed in a specific pathogen-free animal facility in the Second Affiliated Hospital of Harbin Medical University, with a 12-hour light and 12-hour dark cycle. For the generation of ApoE^−/−^RIPK3^−/−^ mice, ApoE^−/−^ mice were mated with RIPK3^−/−^ mice. After genotype identification, ApoE^−/−^RIPK3^−/−^ mice were chosen for further research. Only male mice were used due to the reported influence of estrogen on atherosclerosis progression [21, 22]. The study adhered to the guidelines for experimental atherosclerosis studies described in the American Heart Association Statement [23]. Starting at 8 weeks of age, ApoE^−/−^ and ApoE^−/−^RIPK3^−/−^ mice were fed a high-fat diet containing 21% fat and 0.15% cholesterol (MD12015; Medicine Ltd., China). Mice were randomly divided into two groups (*n* = 20 per group): the control (Ctrl) group and the Tar group with an intraperitoneal injection of cigarette tar (40 mg/kg/day). Blood glucose and body weight are recorded every 2 weeks during the experiments. After 16 weeks, the aortas of the mice were removed after terminal anesthesia (1.5% isoflurane inhalation) and euthanasia by cervical dislocation.

### Pathological assessment

Optimal cutting temperature compound (OCT) or paraffin-embedded hearts were serially sectioned at a thickness of 5 μm to obtain aortic sinus slices. Sections were stained with hematoxylin and eosin (H&E) to analyze plaque morphology, plaque size, and necrotic core area. Oil-Red O (ORO) staining was performed to analyze lipid accumulation in plaques. Masson staining was performed to measure collagen content and fibrous cap thickness. We measured the fibrous cap thickness, which is the minimum distance between the necrotic core and the arterial lumen [24, 25].

### Immunofluorescence and immunohistochemistry staining

Tissue sections were deparaffinized and subjected to antigen retrieval at high temperatures. The membrane was ruptured with 0.1% Triton for 10 minutes after PBS washing and then blocked with 5% bovine serum albumin at room temperature for 1 hr. Then, the tissue sections were incubated with primary antibody at 4 °C in the dark overnight. For immunofluorescence (IF), a fluorescent secondary antibody was used to incubate after being washed with PBS the next day, following incubation with DAPI for 5 min. Images were observed and taken up under a fluorescence microscope (OLYMPUS, Japan), followed by statistical analysis. For immunohistochemistry (IHC), HRP-conjugated secondary antibody and Diaminobenzidine (DAB) were used, and the sections were then counterstained with hematoxylin. Images were obtained using a light microscope (Leica, Germany).

### TUNEL staining

We employed dUTP-digoxigenin incorporation (Roche) for the TUNEL assay. Tissue sections were prepared as previously mentioned in IHC staining and permeabilized with 20 μg/ml of proteinase K. The terminal deoxynucleotidyl transferase (TdT) reaction mix (45 μl equilibration buffer, 5 μl nucleotide mix, and 1 μl TdT enzyme) was prepared during the equilibration step and protected from light. Subsequently, 50 μl of the TdT reaction mix was applied to each slide and incubated in a humid chamber, shielded from light, for 1 hr. at 37 °C. Following a wash with PBS, each slide was incubated with 50 μl POD for 30 min. Finally, tissue sections were incubated with DAB for color development and then counterstained with hematoxylin.

### Cell culture and treatments

Mouse aortic vascular smooth muscle cells (ATCC, USA) were cultured in DMEM medium (Gibco, USA), supplemented with 10% fetal bovine serum (Gibco, USA), and maintained at 37 °C, 95% air/5% CO_2_ in a fully humidified incubator. The cells were treated with trypsin enzyme (Beyotim, China) and seeded in plates for reagent intervention. Cigarette tar (100 μg/ml) was used to stimulate VSMCs.

### Cell viability assays

Cell viability was assessed using enhanced CCK-8 (Dojindo, Japan). Following treatment, 100 μl of CCK-8 working solution was added to each well of a 96-well plate and incubated with VSMCs for 1 hr. at 37 °C before reading the optical density of each well at 450 nm using a microplate reader (Tecan, Switzerland).

### RNA extraction and qRT-PCR

Total RNA was isolated using Trizol regent (Invitrogen, USA) and reverse transcribed into cDNA using the Reverse Transcription kit (Roche, Switzerland). Then, a quantitative real-time polymerase chain reaction qRT-PCR was carried out using SYBR Green (Roche, Switzerland). Each sample was assayed with at least 3 replicates, and the expression of target genes was measured using the 2^−Δ∆Ct^ method. Gene-specific primers were as follows:

*Ripk1-F: 5′-GGGAAGGTGTCTCTGTGTTTC-3′.*

*Ripk1-R: 5′-CCTCGTTGTGCTCAATGCAG-3′.*

*Ripk3-F: 5′-CATAGGAAGTGGGGCTACGAT-3′.*

*Ripk3-R: 5′-AATTCGTTATCCAGACTTGCCAT-3′.*

*Mlkl-F: 5′-AGGAGGCTAATGGGGAGATAGA-3′.*

*Mlkl-R: 5′-TGGCTTGCTGTTAGAAACCTG-3′.*

*β-actin-F: 5′-GTTGAGAACCGTGTACCATGT-3′.*

*β-actin-R: 5′-TTCCCACAATTTGGCAAGAGC-3′.*

### Western blot analysis

Total proteins were extracted using the RIPA lysis buffer (Beyotime, China) containing 1 mM PMSF (Beyotime, China). After measuring and adjusting the protein concentration using a BCA kit (Beyotime, China), the protein samples were mixed with 5x protein loading buffer (Beyotime, China) for electrophoresis. Subsequently, the proteins were transferred to PVDF membranes (Millipore, USA), blocked with 5% skim milk for 1 hr. at room temperature, incubated with a primary antibody at 4 °C overnight, followed by a secondary antibody for 1 hr. at room temperature, and visualized using an ECL Kit (Solarbio, China). The specific antibodies of anti-RIPK1, anti-RIPK3, anti-MLKL, anti-p-MLKL, anti-PERK, anti-p-PERK, anti-GRP78, anti-eIF2α, anti-p-eIF2α, anti-CHOP, anti-p-IP3R, anti-VDAC, anti-CaMKII, anti-p-CaMKII, anti-β-actin, and anti-GAPDH were obtained from Cell Signaling Technology (USA).

### Scanning electron microscopy

Seed cells in a 24-well plate for cell adhesion. After treatment, wash the cells twice with PBS and then soak the glass slides in 2.5% glutaraldehyde solution for preservation at 4 °C. The sample was then treated with 2% uranyl acetate using the potassium chloride conductive method for over 8 hr., washed with double distilled water for 1 hr., dried, sprayed with gold powder, and observed under a scanning electron microscope.

### EdU labeling assay

For the EdU labeling assay, cells were seeded into 96 well plates. After 24 hr. treatment, 10 μmol/L EdU solution was added to each well (RIOBIO, China). The cells were incubated for another 6 hr. at 37 °C, fixed with 4% paraformaldehyde in PBS and stained with a reaction cocktail. DAPI was subsequently used for nuclear staining, followed by imaging with a fluorescence microscope (Leica, Germany).

### Flow cytometry analysis of cell death

After treatment, discard the culture medium and wash the cells three times. Digest the cells with trypsin, centrifuge, and wash them twice with PBS stored at 4 °C. Resuspend the cell pellet, perform a cell count, and adjust the cell density to approximately 1 × 10^6^ cells/ml. Transfer 100 μl of the cell suspension to an EP tube, add 5 μl of Annexin V reagent, and incubate in the dark for 10 min. Add 5 μl of PI reagent, avoid light exposure, and perform the detection within 1 hr.

### Quantitative proteomics and bioinformatics

The total protein from mouse aortas was isolated, and the concentration was measured using BCA for proteomics analysis. 50 μg of protein was taken from each sample and adjusted to the same concentration and volume. Dithiothreitol (DTT) was added to the above protein solution to make the final concentration of DTT 5 mM, and incubated at 55 °C for 30 min. Add the appropriate volume of iodoacetamide to make a final concentration of 10 mM and then 6 times the volume of acetone to the above solution to precipitate the protein at -20 °C overnight. The precipitate was collected by centrifugation and digested overnight at 37 °C with trypsin. Tryptic peptides were vacuum-dried and labeled using Tandem Mass Tags (Thermo, USA) under the manufacturer’s instructions. Peptide fragments were separated by high pH liquid chromatographic and then analyzed by MS. Data were analyzed using Proteome Discover 2.4.1.15 (Thermo, USA). The *p*-values of trusted proteins calculated with Student’s t-test showed significant differences (fold-change ≥1.2 and p-value < 0.05) between groups. The biological function of the proteins was analyzed using KEGG and Gene Ontology databases.

### Measurement of intracellular and mitochondrial Ca^2+^

Intracellular Ca^2+^ and mitochondrial Ca^2+^ levels were measured using Fluo-4, AM (Beyotime, China) and Rhod-2, AM (Maokang, China), respectively, following the manufacturer’s instructions. After treatment, cells were washed three times with PBS and then incubated with the working solution at 37 °C for 30 min. The images were visualized using laser scanning confocal microscopy.

### Mitochondrial membrane potential measurement

JC-1 staining working solution was prepared according to the instructions. The culture medium was removed and washed twice with PBS. Then, the JC-1 staining working solution was added, and the plate was incubated in a cell culture incubator for 20 min. After incubation, the cells were washed twice with the staining buffer and observed under a fluorescence microscope, and pictures were taken. When the cell function is impaired, the JC-1 staining of mitochondrial membrane potential will show a decrease in red fluorescence and an increase in green fluorescence, indicating a loss of mitochondrial membrane potential and potential mitochondrial dysfunction.

### DCFH-DA staining

Dilute the fluorescent probe DCFH-DA with serum-free culture medium to a final concentration of 10 μM. Remove the culture medium and add the diluted DCFH-DA. Incubate the plate in a cell culture incubator for 20 min. After incubation, wash the cells three times with a serum-free culture medium, observe the generation of ROS under a fluorescence microscope, and take pictures.

### PI/Hoechst double staining assay

After treatment, wash the cells in a 96-well plate once. Add 100 μl of staining buffer to each well, followed by 5 μl of Hoechst staining solution and 5 μl of PI staining solution. Mix well and incubate the plate on ice or at 4 °C for 30 min. After staining, wash once with PBS and observe under a fluorescence microscope. The ratio of red to blue fluorescence intensity can be used to quantify cell viability, with a lower ratio indicating a prevalence of live cells and a higher ratio indicating a prevalence of dead cells.

### Co-immunoprecipitations assay

The total protein from the cell lysate was extracted. The extract was incubated with a target antibody or normal rabbit IgG (1:50) control overnight at 4 °C. Then, the extract was incubated with protein G beads for 3 hr. at 4 °C and followed by magnetic pull down. Finally, proteins were eluted by heating at 95 °C for 5 min and analyzed by immunoblotting with antibodies.

### Statistical analysis

All data are reported as the means ± standard deviations. Two-tailed Student’s t-test was used to compare data between two groups with normally distributed values. Mann-Whitney U test was used to compare data between two groups with non-normal distributed values. Statistical significance was set at *P* < 0.05.

## Results

### Tar promoted atherosclerotic plaque progression in ApoE^−/−^ mice

**Fig. 1:**
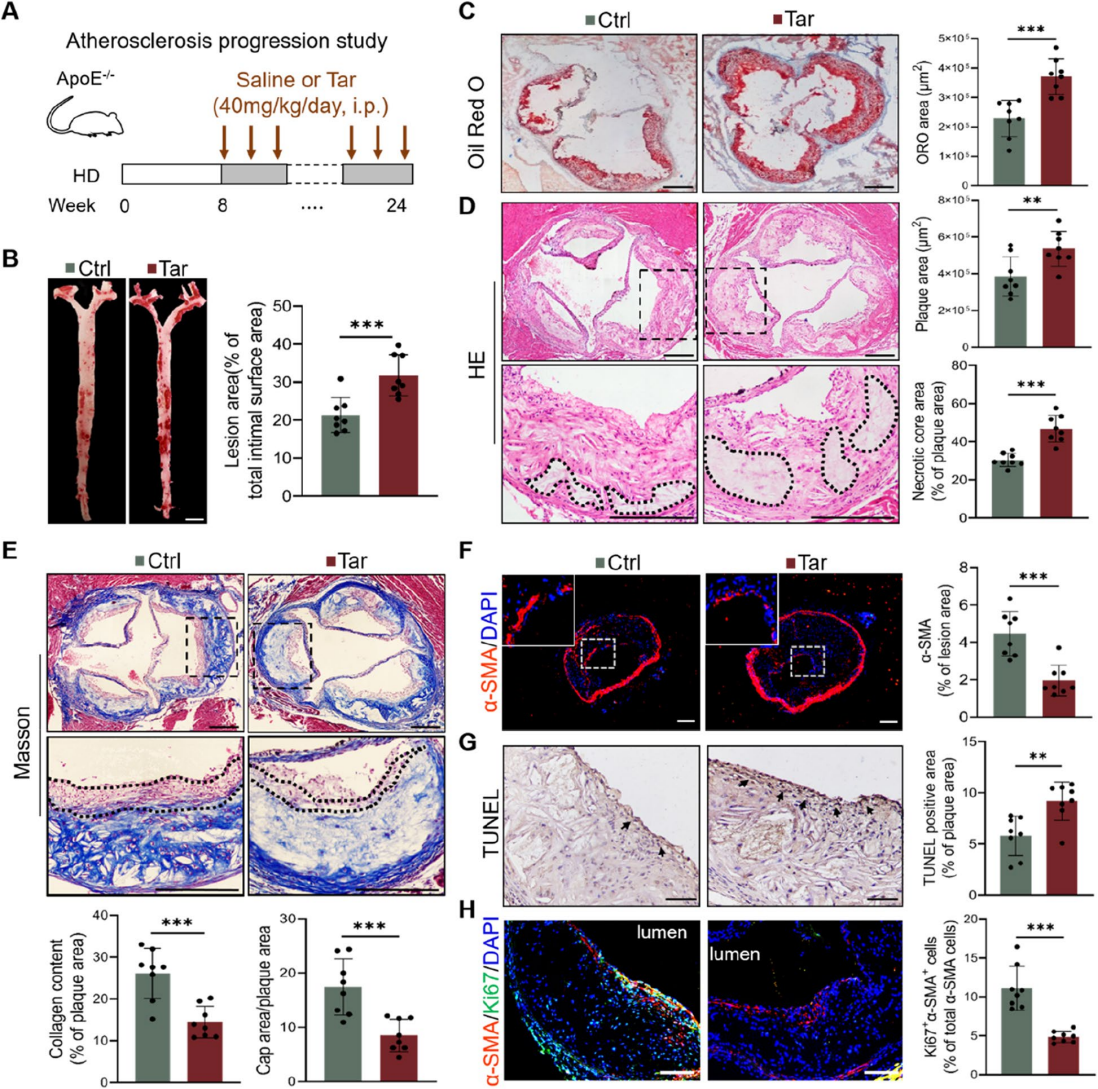
Tar promoted atherosclerotic plaque progression in ApoE^−/−^ mice. **A** Starting at 8 weeks, ApoE^−/−^ mice were fed with high-fat diet and intraperitoneal injected with saline or cigarette tar (40 mg/kg/day) for 16 weeks. *n* = 20. **B** Representative images and quantification of Oil Red O staining in descending aorta. Scale bar: 2 mm. *n* = 8. **C** Representative images and quantification of Oil Red O staining in the aortic root. Scale bar: 200 μm. *n* = 8. **D** Representative images of HE in the aortic root lesions. The position in the dashed rectangular box is enlarged as shown below. Circled by the irregular dotted line is the necrotic core. **E** Representative images of Masson staining. Outlined by the irregular dotted line shows the fibrous cap. *n* = 8. **F** Representative images of α-SMA (VSMCs) immunofluorescence staining in the brachiocephalic artery of mice. The upper left corner is an enlargement of the image in the dashed rectangular. Scale bar: 200 μm. *n* = 8. **G** Representative images of TUNEL staining in aortic root plaques of mice. Scale bar: 100 μm. *n* = 8. **H** Representative images of Ki67 (green), α-SMA (red) staining in the aortic root, and quantification of Ki67^+^α-SMA^+^ cells in total α-SMA^+^ cells. Scale bar: 100 μm. *n* = 8. Data were expressed as mean ± SD. *p < 0.05, **p < 0.01, ***p < 0.001

ApoE^−/−^ mice administrated with either saline or tar (40 mg/kg) intraperitoneally every day were fed high-fat diet for 16 weeks (Fig. 1A). Oil Red O staining on the intact intimal surface of the aortas showed an increase in lipid deposition in Tar group (Fig. 1B). Similarly, tar increased lipid content in the cross-sections of the aorta root (Fig. 1C). Further investigation into potential systemic contributors impacting lesion formation revealed no significant difference in glucose, body weight, food intake, plasma cholesterol, triglycerides, high density lipoproteins (HDL), or low density lipoprotein (LDL) between the control and tar groups (Supplement Fig. 1A-G). Pathological staining indicated that tar not only increased plaque size but also induced marked changes in plaque structural composition. Administration of tar significantly elevated the percentage of necrotic cores (Fig. 1D) and simultaneously lowered the collagen content and fibrous cap area in plaques (Fig. 1E). Moreover, fewer VSMCs per plaque area were detected in the brachiocephalic artery of tar-treated mice (Fig. 1F). In Tar group, TUNEL staining revealed an increase in dead cells at the fibrous cap on the plaque’s surface (Fig. 1G), and the proliferation of VSMCs, detected by the co-localization of Ki67and α-SMA, also decreased (Fig. 1H). The above results indicate that tar accelerates atherosclerotic plaque progression, and its potential mechanism is related to the loss of VSMCs.

### Necroptosis was activated in atherosclerotic plaques of tar-treated mice

**Fig. 2:**
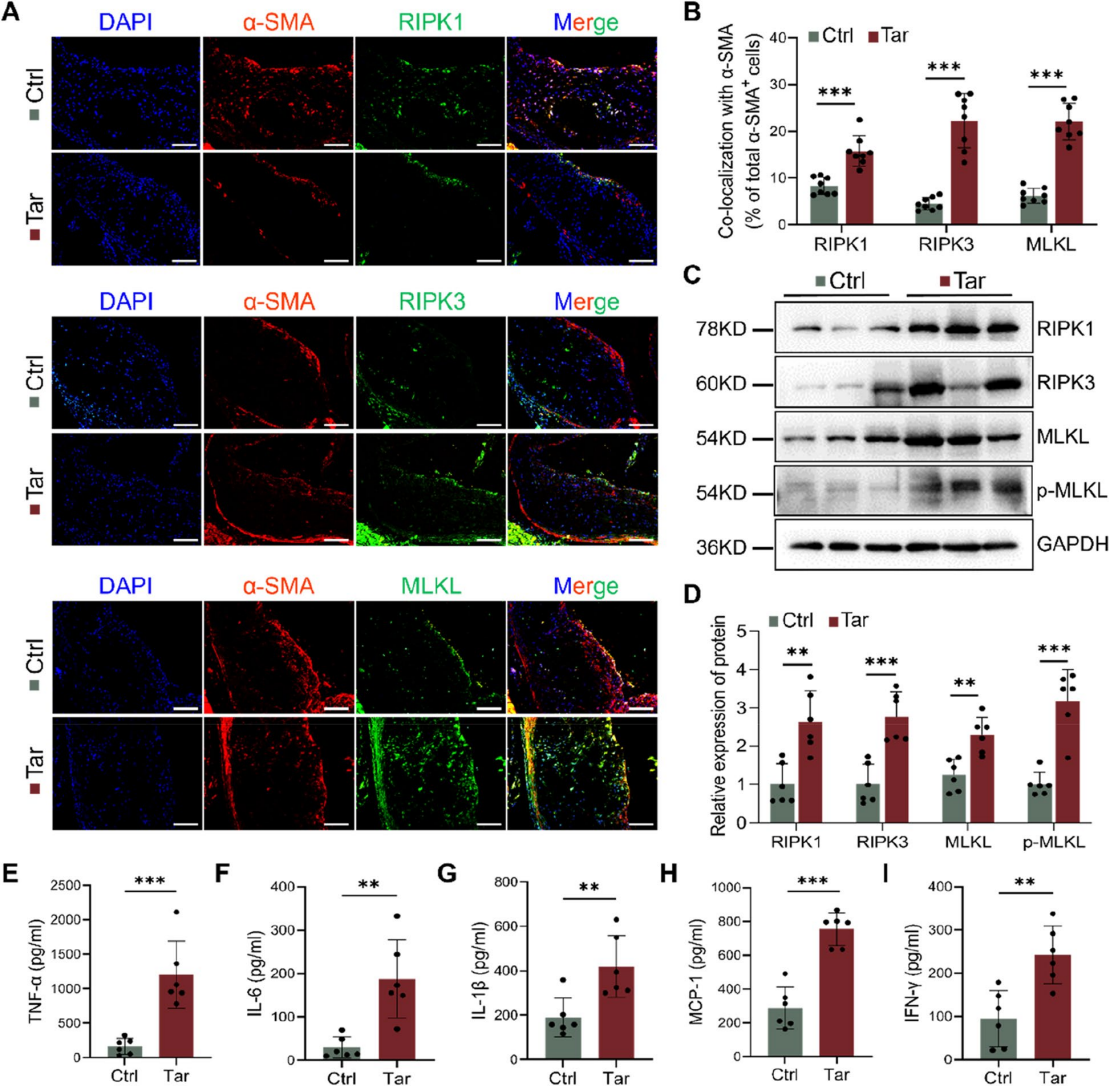
Cigarette tar promotes necroptosis and inflammatory response in ApoE^−/−^ mice. **A-B** Immunofluorescent staining images and quantification of the co-localization of α-SMA and RIPK1, RIPK3, MLKL in aortic roots of mice. Scale bar: 100 μm. *n* = 8. **C-D** Western blot analysis of the protein levels of necroptosis in mice aortas. *n* = 6. **E-I** The levels of inflammatory factors in mice plasm. Data were expressed as mean ± SD. *n* = 6. **p < 0.01, ***p < 0.001

We then investigated the cause of VSMC loss and found that tar significantly increased the expression of necroptotic proteins in VSMCs of plaques (Fig. 2A and B). Western blot results showed that necroptosis was provoked in tar-treated mice aortas (Fig. 2C and D). Necroptotic cells were found to release abundant inflammatory cytokines, so we examined the typical pro-inflammatory cytokine levels in mouse serum. The results indicated a significant elevation of these cytokines in the Tar group (Fig. 2E-I). Similar findings were observed in the carotid artery plaques of smokers. The key protein and mRNA levels of necroptosis were notably higher in smokers compared to non-smokers (Supplement Fig. 2A and B). Immunohistochemical staining revealed that plaques from smokers exhibited more positive staining in close proximity to the fibrous cap (Supplement Fig. 2C). Additionally, RIPK1, RIPK3, and MLKL were found to be elevated in VSMCs of carotid plaques in smokers (Supplement Fig. 2D).

### Tar induces VSMC necroptosis in a RIPK3-dependent way

**Fig. 3:**
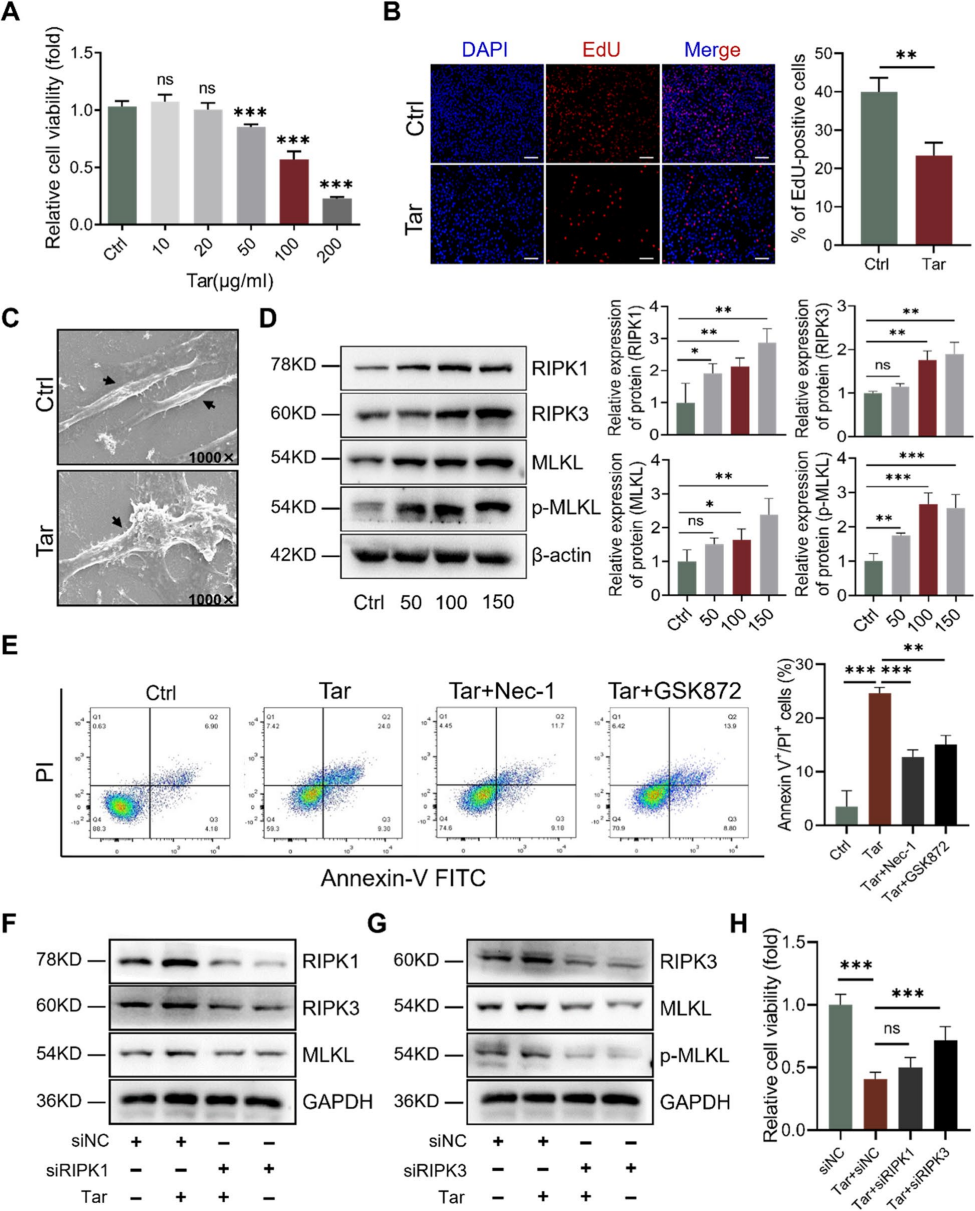
Cigarette tar induces necroptosis in VSMCs. **A** Relative cell viability of VSMCs treated with various concentrations of tar for 24 hr. **B** Representative images and quantification of EdU staining to evaluate the proliferation of VSMCs. Scale bar: 50 μm. **C** Representative images of scanning electron microscopy of VSMCs treated with or without tar (100 μg/ml) for 24 hr. The black arrows indicate VSMCs. **D** Western blot analysis of the protein levels of necroptosis treated with various concentrations of tar in VSMCs. **E** Flow cytometry analysis of Annexin-V/PI fluorescence in VSMCs treated with tar (100 μg/ml) with or without necroptosis inhibitor. Nec-1 (10 μM), the specific inhibitor of necroptosis. GSK872 (10 μM), the specific inhibitor of RIPK3. **F** and **G** Representative immunoblots of necroptotic proteins treated with tar after knockdown RIPK1 (**F**) or RIPK3(**G**) by siRNA. *n* = 3. **H** Relative cell viability of VSMCs treated with tar after knockdown RIPK1 or RIPK3 by siRNA. Data were expressed as mean ± SD. *p < 0.05, **p < 0.01, ***p < 0.001

To further understand the effect of tar-induced VSMC necroptosis, we conducted an in vitro study applying cigarette tar. As shown in Fig. 3A, tar depressed VSMC cell viability in a concentration-dependent manner, with relative cell viability falling to about 50% at a concentration of 100 μg/ml. EdU staining also demonstrated decreased VSMC proliferation due to tar (Fig. 3B). Scanning electron microscopy imaging showed that VSMCs swelled and ruptured when treated with tar (Fig. 3C), consistent with the morphological characteristics of necroptosis. Moreover, western blot analysis showed that tar significantly upregulated the expression of RIPK1, RIPK3, MLKL, and p-MLKL (Fig. 3D). Nec-1 and GSK872, specific inhibitors of necroptosis and RIPK3 respectively, were used to pretreat VSMCs to verify the activation of necroptosis by tar. Flow cytometry analysis of Annexin V/PI staining exhibited that tar-induced cell death rate was attenuated by pretreatment with Nec-1 or GSK872 (Fig. 3E). Whereas the inhibitors of other types of programmed cell death (zVAD, VX765, and Fer1) had no effect on tar-induced VSMC death (Supplement Fig. 3).

We then silenced RIPK1 and RIPK3, respectively, in VSMCs to investigate whether the induction of necroptosis by tar is dependent on these proteins. The western blot results showed that MLKL and p-MLKL were significantly downregulated after being transfected with siRIPK1 and siRIPK3 (Fig. 3F and G). Interestingly, it was observed that the decreased cell viability induced by tar was improved after being transfected with siRIPK3, whereas there was no remarkable effect of siRIPK1 in protecting cell viability (Fig. 3H). These data indicated that cigarette tar induced VSMCs to undergo necroptosis in a RIPK3-dependent way.

### RIPK3 deletion ameliorated atherosclerosis progression caused by tar

**Fig. 4:**
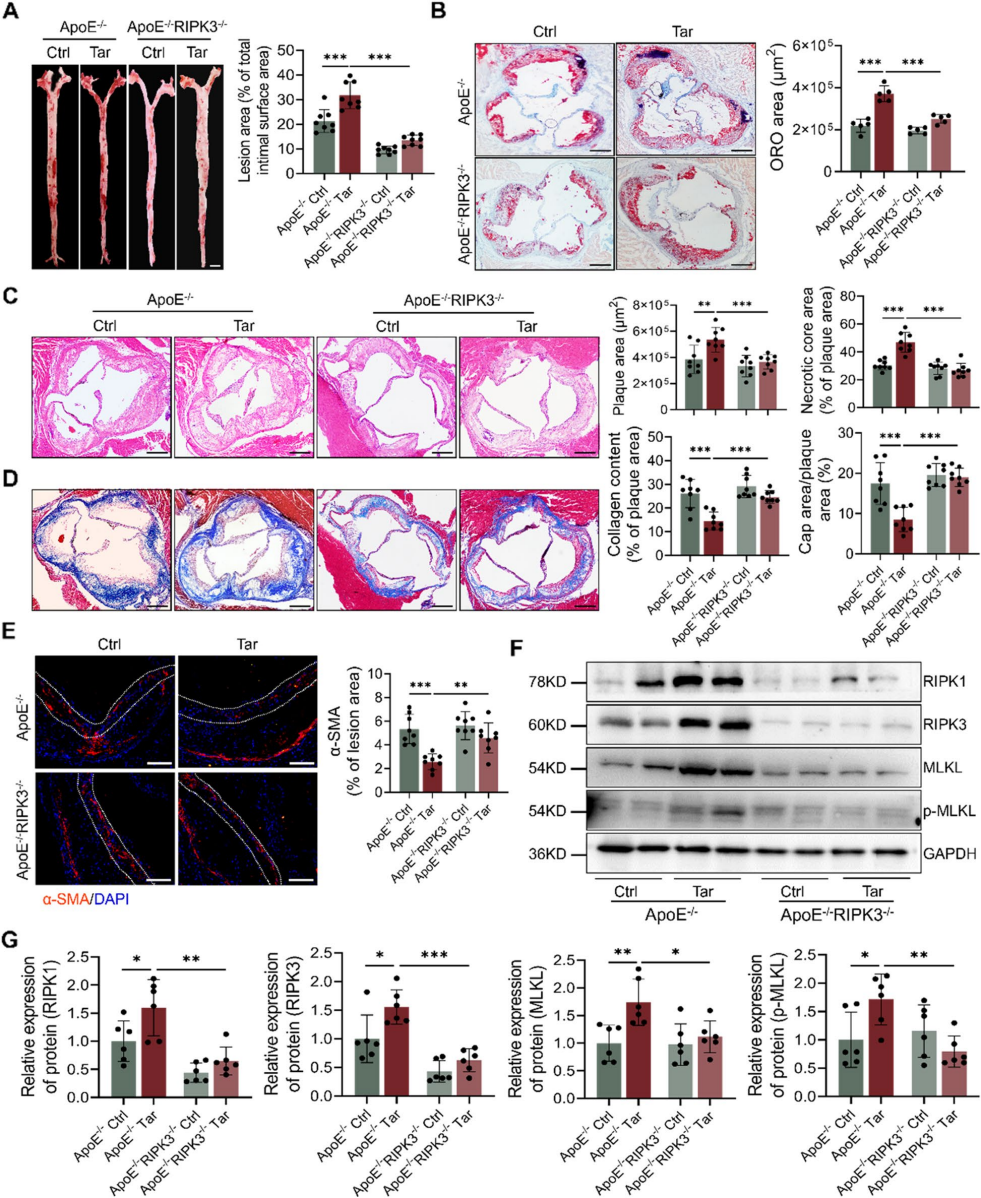
RIPK3 deletion ameliorated atherosclerosis progression caused by cigarette tar. **A** Representative images and quantification of Oil Red O staining in descending aorta. Scale bar: 2 mm. *n* = 8. **B** Representative images and quantification of Oil Red O in aortic roots. Scale bar: 200 μm. *n* = 5. **C** HE staining and quantification of plaque area and necrotic core area in aortic roots. Scale bar: 200 μm. *n* = 8. **D** Masson staining and quantification of collagen content and the percent of fibrous cap area in aortic roots. Scale bar: 200 μm. *n* = 8. **E** α-SMA (VSMCs) immunofluorescence staining and the percentage of it in the total lesion area. The position between the double dashed lines indicated the fibrous cap. Scale bar: 100 μm. *n* = 8. **F-G** Western blot analysis necroptosis protein levels. *n* = 6. Data were expressed as mean ± SD. *p < 0.05, **p < 0.01, ***p < 0.001

To investigate the role of RIPK3-dependent necroptosis in tar-induced atherosclerotic lesion progression, ApoE^−/−^RIPK3^−/−^ mice were utilized. Enface analysis of Oil Red O staining revealed that ApoE^−/−^RIPK3^−/−^ mice exhibited a significantly reduced lesion area compared to ApoE^−/−^ mice treated by tar (Fig. 4A). Similarly, RIPK3 deletion notably diminished tar-increased atherogenesis in the aortic roots (Fig. 4B).

In addition, ApoE^−/−^RIPK3^−/−^ mice exposed to tar displayed markedly decreased plaque area and necrotic core area in the aortic roots compared to tar-exposed ApoE^−/−^ mice (Fig. 4C). Collagen content and fibrous cap areas were also enhanced in ApoE^−/−^RIPK3^−/−^ mice exposed to tar (Fig. 4D). Moreover, ApoE^−/−^RIPK3^−/−^ mice exposed to tar exhibited improved α-SMA expression in the plaque area compared to ApoE^−/−^ mice (Fig. 4E). Deletion of RIPK3 also blocked the necroptosis pathway and inhibited tar-induced MLKL and p-MLKL protein expression (Fig. 4F and G). The expression of RIPK1 and MLKL in VSMCs was also reduced by RIPK3 deletion (Supplement Fig. 4A-D). These findings confirmed that intervening in necroptosis suppressed VSMC loss and atherosclerosis progression caused by tar.

### ER stress is involved in the regulation of tar-induced necroptosis

**Fig. 5:**
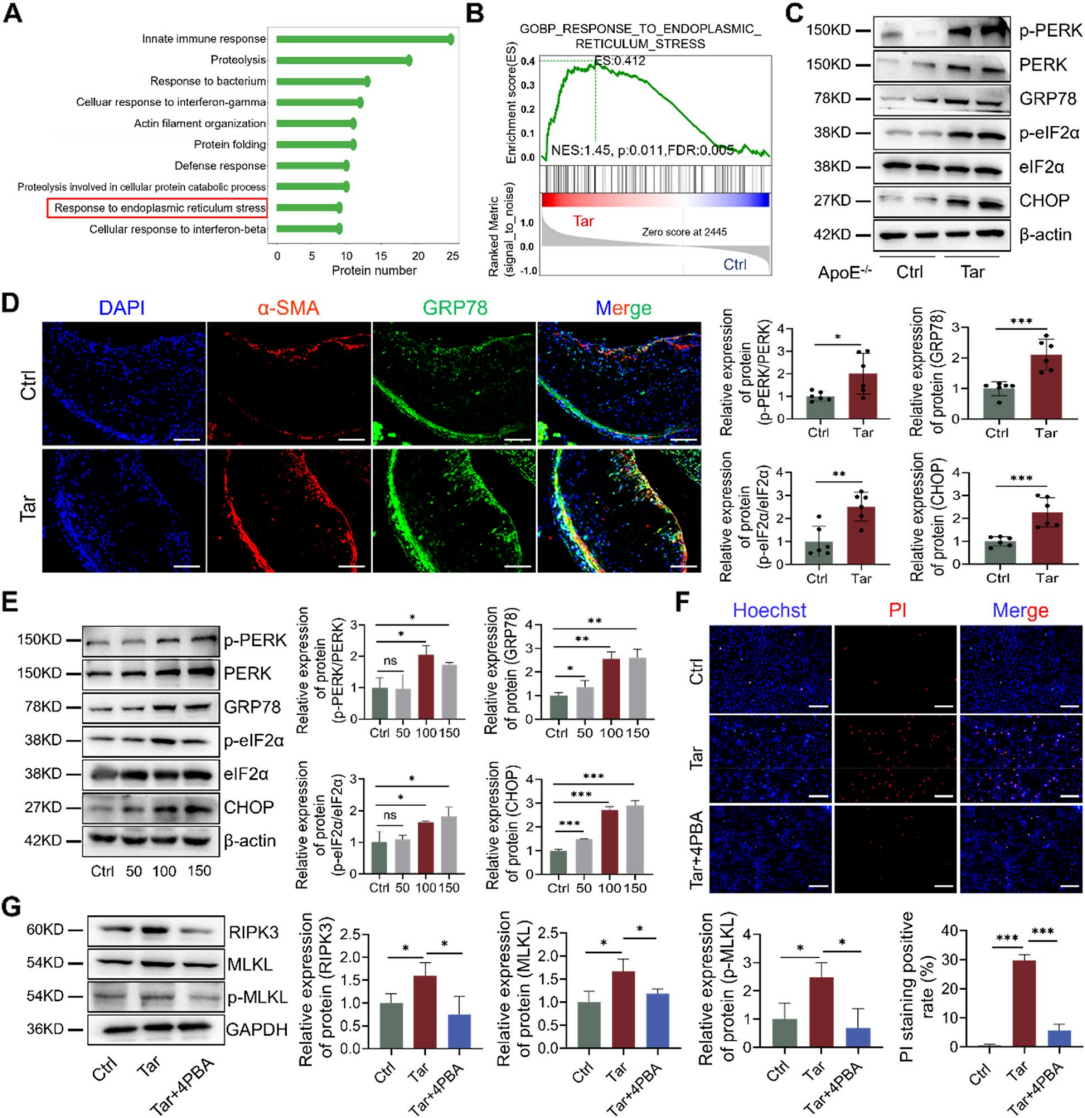
ER stress is involved in the regulation of tar-induced necroptosis. A-B. Quantitative proteomic and bioinformatic analysis of aortas from ctrl and tar-treated mice. **A** Top 10 significantly enriched gene ontology terms in biological process. **B** GSEA result of “response to ER stress”. *P* values and the normalized enrichment score (NES) are presented. **C** ER stress proteins (p-PERK, PERK, GRP78, p-eIF2α, eIF2α, and CHOP) in mouse aortas. *n* = 6. **D** Representative immunofluorescence images of VSMCs (α-SMA; red), ER stress marker GRP78 (green), and DAPI (blue) in aortic roots. Scale bar: 100 μm. **E** Western blot analysis of ER stress protein levels in cultured VSMCs. **F** Hoechst/PI staining indicates the cell death rate of VSMCs treated by tar with or without the ER stress inhibitor 4PBA (20 μM). Scale bar: 50 μm. **G** Western blot analysis necroptosis proteins in VSMCs treated by tar with or without 4PBA. *n* = 3. Data were expressed as mean ± SD. ^*ns*^p > 0.05, *p < 0.05, **p < 0.01, ***p < 0.001

To further explore the regulatory mechanism of tar-induced VSMC necroptosis, we conducted quantitative proteomics experiments using tandem mass tag (TMT) labeling. Among the top 10 enriched GO biological processes associated with significantly altered proteins between Ctrl and Tar groups, proteins involved in ER stress were significantly activated (Fig. 5A). Further gene set enrichment analysis (GSEA) revealed that proteins responding to ER stress were consistently more abundant in the Tar group than in the Ctrl group. This suggests that ER stress, at least in part, contributed to the plaque progression induced by tar (Fig. 5B). Western blot results showed that the PERK-eIF2α-CHOP axis was activated and the ER stress marker GRP78 was significantly upregulated in tar-treated mice (Fig. 5C). Moreover, immunofluorescence staining indicated GRP78 expression in VSMCs was increased in the aortic roots of tar-treated mice compared with control mice (Fig. 5D).

In vitro, significantly elevated protein expression levels of GRP78, p-PERK, p-eIF2α, and CHOP were found in VSMCs treated with tar (Fig. 5E). Subsequently, we applied 4PBA, an inhibitor of ER stress, to pretreat VSMCs, and it was found that 4PBA ameliorated the cell viability suppressed by tar (Fig. 5F). Meanwhile, 4PBA obviously dampened tar-induced upregulation of RIPK3, MLKL, and p-MLKL (Fig. 5G). Taken together, all these findings suggested that tar evoked ER stress, which may account for the necroptosis of VSMCs.

### ER stress inhibition alleviates tar-induced atherosclerosis progression

**Fig. 6:**
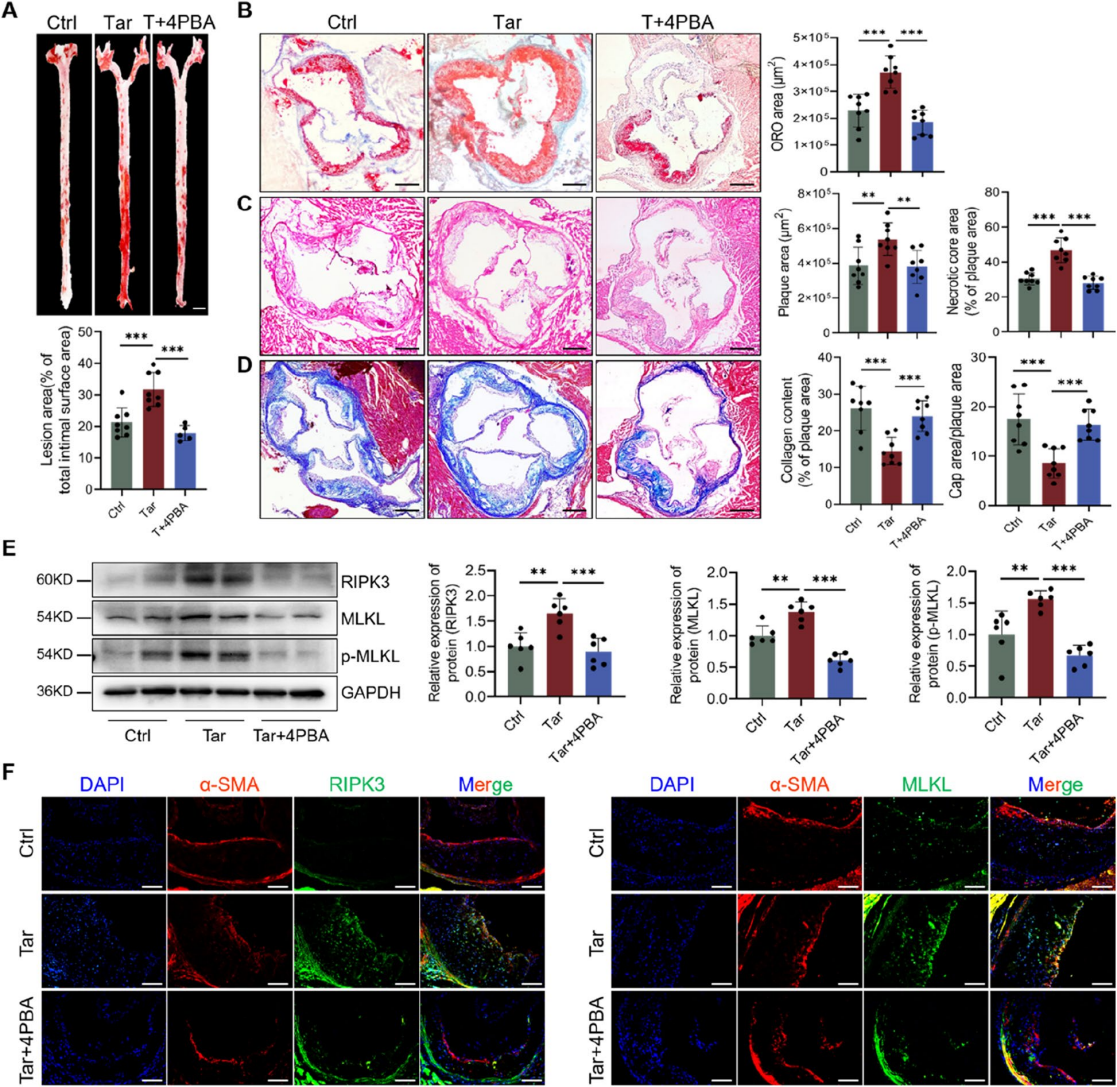
ER stress inhibition alleviates tar-induced atherosclerosis progression. For the ER stress inhibition experiment, 8-week-old male ApoE^−/−^ mice were treated with or without 4PBA (100 mg/kg every other day) by intraperitoneal injection immediately after tar-infusion and for the following 16 weeks. **A** Representative images and quantification of Oil Red O staining in descending aorta. Scale bar: 2 mm. **B** Representative images of Oil Red O staining at aortic roots, scale: 200 μm. **C **HE staining and quantification of plaque area and necrotic core area in aortic roots. Scale bar: 200 μm. **D** Masson staining and quantification of collagen content and the percent of fibrous cap area in aortic roots. Scale bar: 200 μm. **E** The protein expression of RIPK3/MLKL/p-MLKL in mice aortas. **F** α-SMA and RIPK3/MLKL co-staining in aortic roots. Scale bar: 100 μm. Data were expressed as mean ± SD. *p < 0.05, **p < 0.01, ***p < 0.001

To determine the role of ER stress in tar-induced atherosclerosis progression, we treated mice with 4PBA, an ER stress inhibitor. As shown in Fig. 6A, 4PBA significantly reduced the lipid deposition on the intact aorta’s intimal surface. Consistent with this finding, the proportion of ORO-positive areas in the root cross-sections of the aortas was reduced by 4PBA (Fig. 6B). The ER stress inhibitor not only reduced the atherosclerotic plaque area in ApoE^−/−^ mice enhanced by tar, but also markedly decreased tar-augmented necrotic core area (Fig. 6C). In addition, mice treated with 4PBA exhibited increased collagen content and fibrous cap area, compared with the Tar group (Fig. 6D). Furthermore, 4PBA not only suppressed the expression of RIPK3/MLKL/p-MLKL in aortas (Fig. 6E), but also decreased RIPK3 and MLKL in VSMCs of the aortic sinus (Fig. 6F). These data indicate that inhibiting ER stress substantially prevents tar-exacerbated atherosclerosis formation and progression by regulating VSMC necroptosis.

### Mitochondrial Ca^2+^ overload induced by ER stress leads to increased mtROS release and provokes necroptosis in VSMCs

**Fig. 7:**
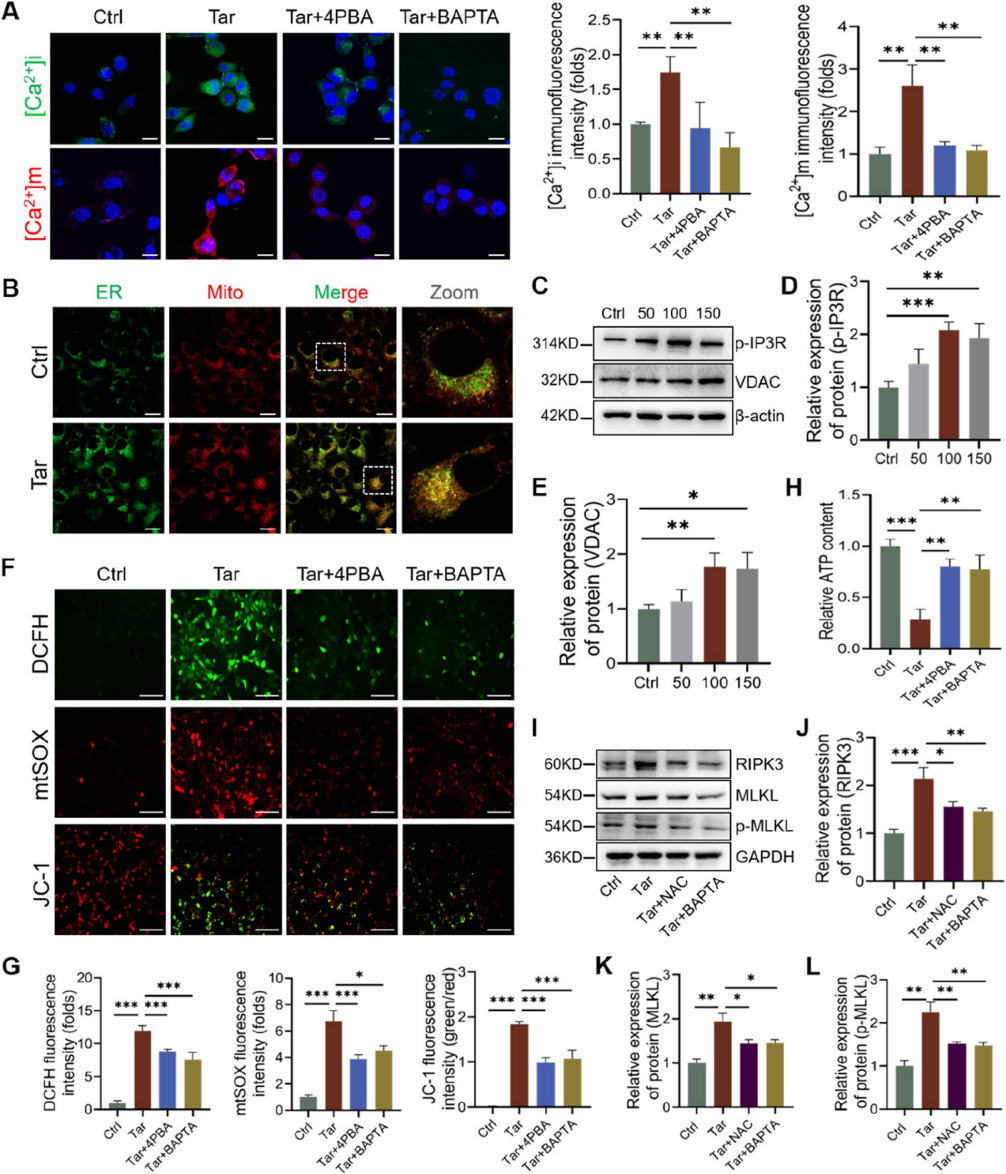
Mitochondrial Ca^2+^ overload induced by ER stress leads to increased mtROS release and provokes necroptosis in VSMCs. **A** VSMCs were pretreated with 4PBA (20 μM) or calcium ion chelator (10 μM) and then intracellular calcium ([Ca^2+^]i) and mitochondrial calcium ([Ca^2+^]m) are stained using Fluo-4 AM and Rhod-2, respectively. Scale bar: 20 μm. **B** Representative staining of ER tracker and Mitotracker. Scale bar: 20 μm. **C-E** Western blot analysis of p-IP3R and VDAC. **F** and **G** Reactive oxygen species (ROS), mitochondrial ROS, and mitochondrial membrane depolarisation level are analyzed by DCFH, mtSOX, and JC-1 respectively in the indicated groups. Scale bar: 100 μm. **H** ATP levels in VSMCs were detected by ATP assay kit in the indicated groups. **I-L** Western blot analysis of necroptosis proteins pretreated with ROS scavenger NAC (5 mM) and BAPTA. *n* = 3. Data were expressed as mean ± SD. *p < 0.05, **p < 0.01, ***p < 0.001

Ca^2+^ plays a fundamental role in cell life, modulating several pathways, such as proliferation, cell death, and survival. The ER is the primary intracellular Ca^2+^ reservoir, and ER stress causes an imbalance between Ca^2+^ release and uptake mechanisms, inducing Ca^2+^ overload. In this study, immunofluorescence of Ca^2+^ staining detected a rise in both intracellular calcium ([Ca^2+^]i) and mitochondrial calcium ([Ca^2+^]m) in tar-treated VSMCs, which could be reversed by 4PBA or the Ca^2+^ chelator BAPTA (Fig. 7A). Regulated transfer of Ca^2+^ from the ER to mitochondria occurs mainly through the opening of voltage-dependent anion channel (VDAC)/inositol 1,4,5-triphosphate receptor (IP3R) complex in the mitochondria-associated endoplasmic reticulum membranes (MAMs). We used ER and mitochondria probes to observe the formation of MAMs, and the results showed that the two organelles were located closer after tar treated (Fig. 7B). Western blot analysis showed that the expression of p-IP3R and VDAC were upregulated in the Tar group (Fig. 7C-E). These data suggest that ER stress may enhance mitochondria uptake of Ca^2+^ through MAMs and result in [Ca^2+^]m overload.

Then, we used fluorescent probes DCFH and mtSOX to measure the repertoire of free radicals in tar-treated VSMCs, and the signals from both probes were significantly higher than those of the Ctrl group (Fig. 7F and G). JC-1 staining also showed that mitochondrial membrane potential in VSMCs was repressed by tar (Fig. 7F and G). Additionally, the production of ATP was found to be dampened by tar (Fig. 7H). However, 4PBA and BAPTA rescued all these tar-induced detrimental effects of mitochondrial homeostasis (Fig. 7F-H). Then, we observed that the ROS scavenger NAC and BAPTA prevented tar-induced RIPK3, MLKL, and p-MLKL protein expression (Fig. 7I-L). These data suggest that [Ca^2+^]m overload causes mitochondrial dysfunction and increased ROS production, which induces RIPK3-dependent necroptosis in VSMCs eventually.

### Cytoplasmic Ca^2+^ overload induces RIPK3-dependent necroptosis via CaMKII

**Fig. 8:**
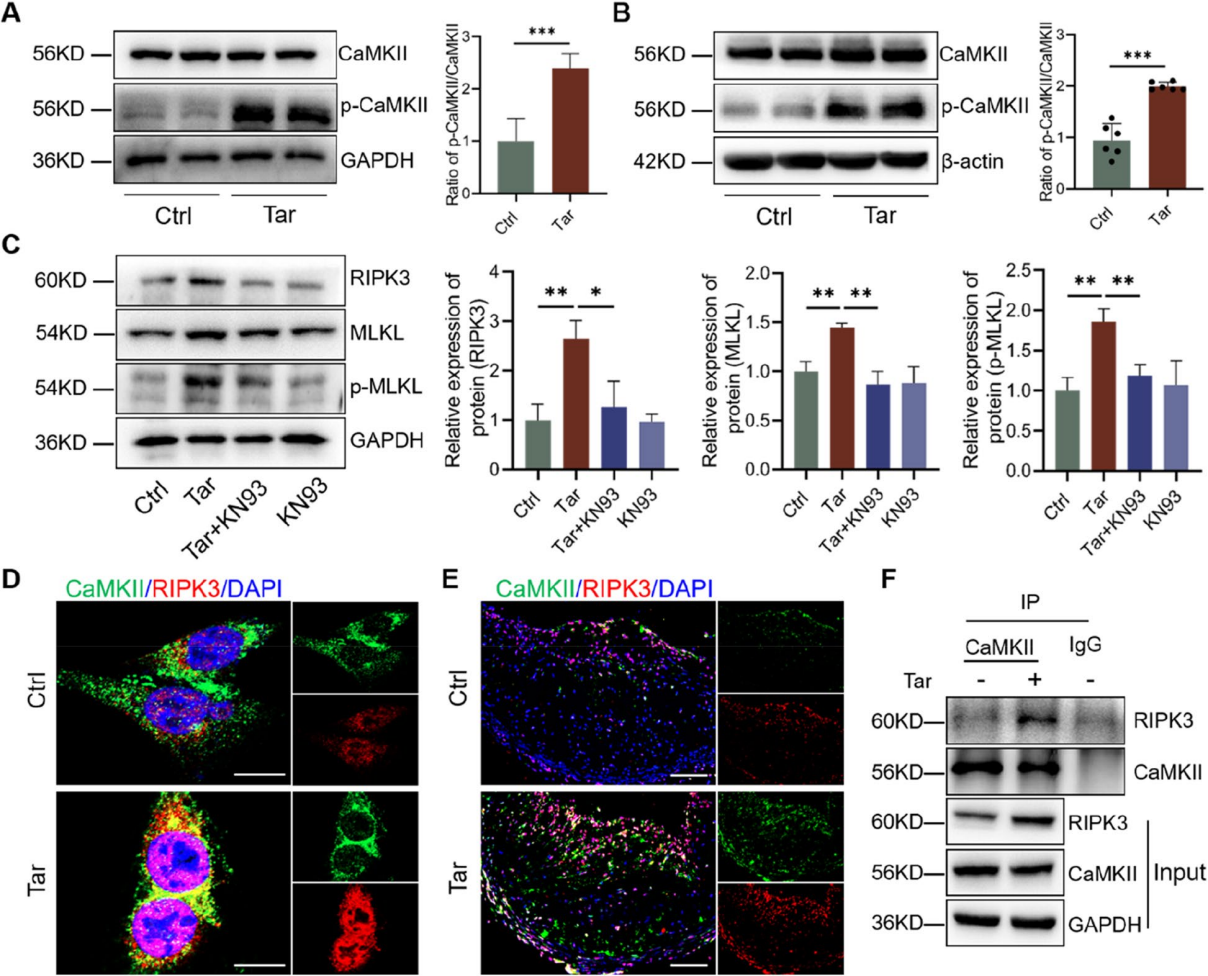
Cytoplasmic Ca^2+^ overload induces RIPK3-dependent necroptosis via CaMKII. **A** Western blot analysis of CaMKII and p-CaMKII in VSMCs. **B** Western blot analysis of CaMKII and p-CaMKII in mice aortas. **C** The expression of necroptosis proteins in VSMCs treated by tar with or without the inhibitor of CaMKII KN93 (10 μM). **D** Double immunofluorescence staining of RIPK3 and CaMKII in VSMCs. Scale bar: 20 μm. **E** Representative immunofluorescent staining images of the co-localization of CaMKII and RIPK3 in the aortic root of mice. **F** Cell lysates from VSMCs untreated or treated with tar were subject to immunoprecipitation (IP) by IgG or anti-CaMKII antibody, followed by western blotting for RIPK3 and CaMKII. Scale bar: 100 μm. *n* = 8. Data were expressed as mean ± SD. *p < 0.05, **p < 0.01, ***p < 0.001

CaMKII is the primary responder and regulator of intracellular Ca^2+^ signaling. Here, we tested the effect of tar on CaMKII. The results in VSMCs showed that the expression of p-CaMKII was upregulated by tar (Fig. 8A). Similarly, tar increased the expression of p-CaMKII in aortas (Fig. 8B). Using the NetPhos 3.1 Server (http://www.cbs.dtu.dk/services/NetPhos/, Technical University of Denmark), we found that CaMKII is a potential upstream kinase of mouse RIPK3, which binds and phosphorylate RIPK3, inducing necroptosis eventually. To further explore the regulatory relationship between CaMKII and necroptosis, we used KN93, an inhibitor of CaMKII, to pretreat VSMCs, and the results showed that KN93 decreased tar-induced upregulation of RIPK3, MLKL, and p-MLKL significantly (Fig. 8C). In addition, cytoplasmic CaMKII merged well with RIPK3 in cultured VSMCs and aortic root plaques of the Tar group (Fig. 8D and E). The anti-CaMKII antibody pulled down markedly more RIPK3 proteins in tar-treated VSMCs, indicating that tar causes CaMKII to physically associate with RIPK3 (Fig. 8F).

## Discussion

**Fig. 9:**
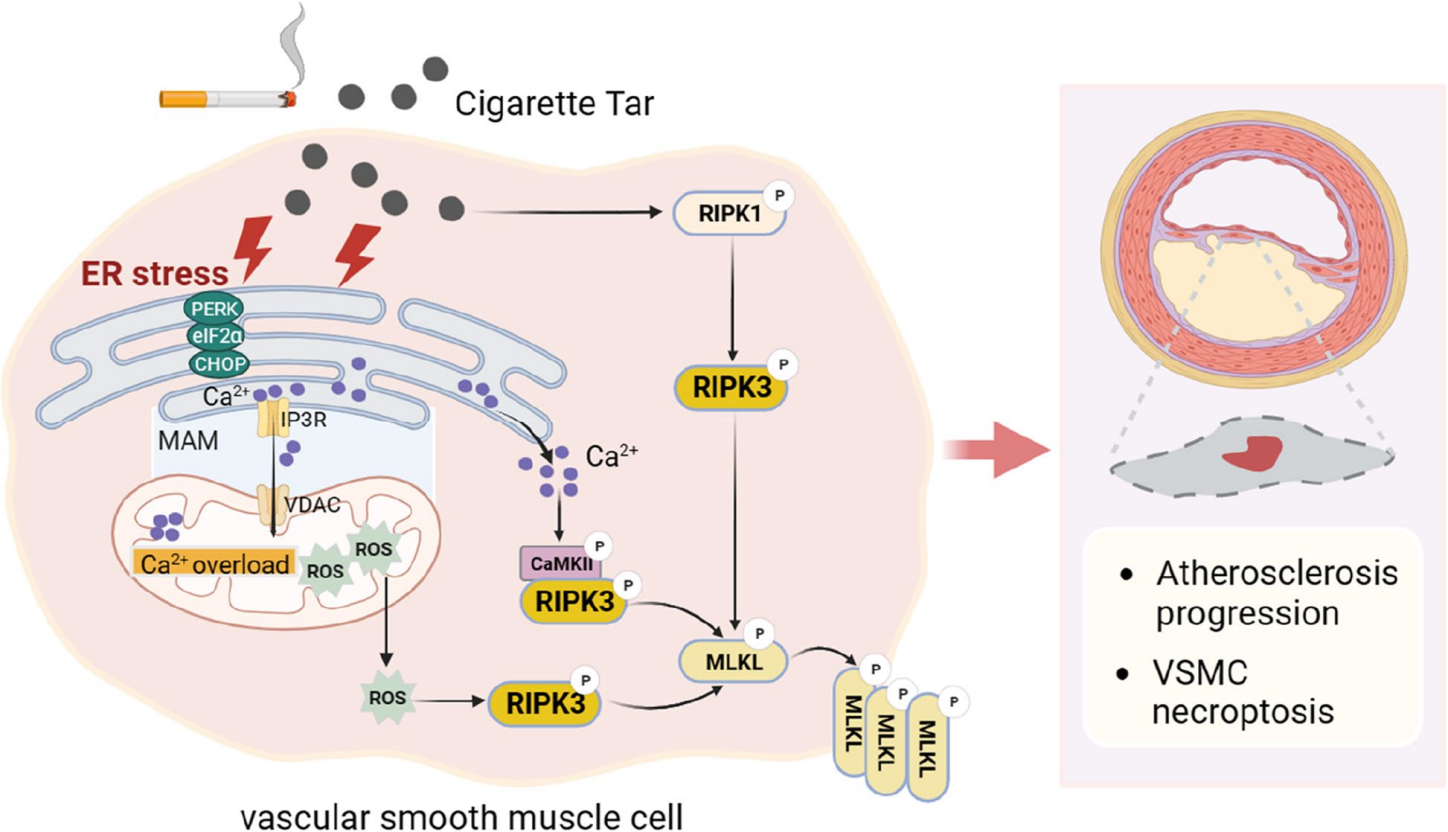
Graphic abstract of the underlying molecular mechanism of tar-induced atherosclerosis progression

In this study, we systemically examined the crucial role of cigarette tar in atherosclerosis progression and made several novel discoveries. Firstly, we found that cigarette tar causes VSMC necroptosis, thereby accelerating the advancement of atherosclerosis for the first time. Secondly, we determined that tar-induced ER stress plays a pivotal role in VSMC necroptosis and the progression of atherosclerosis. Lastly, we clarified that ER stress mediates tar-induced RIPK3-dependent necroptosis through mitochondrial ROS and the Ca^2+^/CaMKII pathway (Fig. 9).

Multiple studies have demonstrated that smoking independently contributes to various cardiovascular diseases. While there has been extensive research into the mechanisms of atherosclerosis associated with smoking, particularly focusing on nicotine, no approach effectively shields individuals exposed to cigarette smoking from developing atherosclerosis [26, 27]. Our data illustrated that cigarette tar exacerbates atherosclerosis, which was attenuated by genetic deletion of RIPK3. It’s noteworthy that the nicotine concentration used in previous studies was approximately 10 times higher than that found in the plasma of chronic smokers [28–30]. This might be due to the fact that low doses of nicotine are less toxic but are mainly associated with sustaining smokers’ addiction [31]. What’s more, a general strategy for reducing the harm of smoking has been proposed: “retaining nicotine at pleasurable or addictive levels while reducing tar” [32]. This indicates that the role and mechanism of tar in atherosclerosis deserve more attention. Our findings extend the current knowledge of the effect and mechanism by which smoking promotes plaque progression.

Necroptosis, a newly identified programmed cell death, has been found to be involved in several smoking-related diseases. Hannelore et al. revealed that cigarette smoke exposure induced RIPK1-dependent inflammation and cell death, contributing to the pathogenesis of chronic obstructive pulmonary disease [33]. Zhe et al. also found that the protein levels of RIPK3 and MLKL in epithelium and macrophages of lung tissue were increased in cigarette smoke-exposed mice [17]. Notably, the involvement of classical necrosome “RIPK1/RIPK3/MLKL” was an indication but not a requirement to trigger necroptosis. In our study, we confirmed that tar induced VSMC necroptosis and the increased death rate can be improved by specific inhibitors. However, siRNA targeting RIPK3 but not RIPK1 reversed the suppressed cell viability caused by tar. This is likely because RIPK1 is also involved in cell survival and can lead cells to divergent outcomes through various signaling pathways stimulated by different factors [34]. Regarding RIPK3, it can be activated in a RIPK1-independent manner and subsequently execute necroptosis by phosphorylating MLKL. For instance, it has been demonstrated that Smad3 was capable of binding to RIPK3 and MLKL promoter regions, and this physical binding significantly enhanced the Smad3-RIPK3/MLKL necroptosis pathway in diabetic mice [35]. mTOR and SLC9A1 have also been found to stimulate necroptosis through the direct activation of the kinase activity of RIPK3 [36, 37]. Taken together, these findings provide new mechanistic insights into tar-induced atherosclerosis progression and identify RIPK3 as a potential therapeutic target to prevent atherosclerosis in individuals who have not successfully quit smoking.

Another major finding of the present study is that we demonstrated ER stress as a key regulator in tar-induced VSMC necroptosis. Accumulating studies have reported that ER stress is involved in the progression of atherosclerosis [20, 38, 39]. Persistent ER stress ultimately leads to cell death. Lugea et al. discovered that cigarette smoke induces cell death by inhibiting the adaptive unfolded protein response signaling pathway and activating ER stress pathways, leading to acinar cell death [40]. In addition, it has been reported that ER stress could induce necroptosis in L929 cells [41]. However, the role of VSMC ER stress in mediating necroptosis and atherosclerosis remains unclear. It has been reported that there are three transmembrane protein kinases in the ER membrane involved in ER stress, the protein kinase R (PKR)-like ER kinase (PERK), the kinase and endoribonuclease (IRE1), and the basic leucine-zipper activating transcription factor 6 (ATF6) [42]. PERK, when activated by phosphorylation, can promote apoptosis by inducing the expression of CHOP, known for its potent pro-apoptotic pathways [43]. In our study, Grp78, p-PERK, p-eIF2α, and CHOP were highly expressed within plaques and VSMCs in the Tar group, suggesting that tar might induce ER stress to activate the PERK/eIF2α/CHOP pathway. The ER stress inhibitor 4PBA attenuated VSMC necroptosis and ameliorated atherosclerosis progression. We report for the first time that ER stress was significantly activated in tar-triggered VSMC necroptosis, consequently advancing atherosclerosis progression.

Regarding the downstream signaling mechanism, ER stress induces a disturbance of Ca^2+^ homeostasis, leading to the accumulation of Ca^2+^ in both mitochondria and cytoplasm, exerting deleterious effects [44, 45]. Mitochondrial Ca^2+^ overload induces ROS overproduction by causing complex II disintegration, resulting in the opening of the mitochondrial permeability transition pore and the release of ROS [46, 47]. Weindel et al. found that elevated mitochondrial ROS promoted macrophage necroptosis in a RIPK3/MLKL-dependent manner [48]. In this study, we observed the accumulation of [Ca^2+^]_m_, resulting in mitochondrial dysfunction and excessive ROS production. Notably, necroptosis was markedly downregulated after scavenging excess ROS and Ca^2+^. Mitochondria uptake Ca^2+^ from the cytoplasm or adjacent organelles [49]. MAMs, the juxtaposition of ER and mitochondria, mediate ER-mitochondria Ca^2+^ transfer [50, 51]. In our study, we observed excessive coupling of mitochondria and ER and increased expression of VDAC and p-IP3R, triggering excess mitochondrial Ca^2+^ entry in VSMCs. These findings revealed the mechanisms regulating the interplay between ER stress and tar-induced necroptosis.

CaMKII is a serine/threonine protein kinase with a wide range of functions [52]. Elevated intracellular Ca^2+^ concentration allows Ca^2+^ to bind and activate CaMKII [53]. Given the result that tar induced Ca^2+^ overload, we further found that tar increased the phosphorylated CaMKII in cultured VSMCs and aortas. In fact, activated CaMKII has been observed in advanced human and mouse plaque macrophages, contributing to plaque instability [54]. Zhang et al. confirmed that RIPK3 can directly bind to CaMKII in oxidative stress-induced myocardial necroptosis, with CaMKII serving as a downstream protein of RIPK3 in their study [55]. In our research, co-immunoprecipitation experiments also detected an interaction between RIPK3 and CaMKII during VSMC necroptosis. However, we demonstrated that inhibiting CaMKII reduced tar-induced upregulation of RIPK3, MLKL, and p-MLKL in VSMCs, suggesting that CaMKII may act as an upstream kinase of RIPK3. The discrepancy between our results and previous literature may stem from two main factors. Firstly, CaMKII can phosphorylate and regulate various downstream pathways affecting gene expression, inflammation, metabolism, and cell death [56]. Zhang et al. recently found that CaMKII impairs DNA repair by phosphorylating and promoting the degradation of ubiquitin-conjugating enzyme E2T [57]. Additionally, the role of CaMKII in regulating RIPK3 has not been explored previously. Our results for the first time suggest that CaMKII can regulate RIPK3 activation. To further verify our results, we used protein phosphorylation site prediction and found that CaMKII can serve as an upstream kinase of RIPK3, leading to its phosphorylation and activation. Nevertheless, additional in vitro and in vivo experiments are necessary to confirm these results.

## Conclusion

Our study demonstrated that cigarette tar accelerated the progression of atherosclerotic plaque. We proposed a novel mechanism through which tar induced VSMC necroptosis in a RIPK3-dependent way, via a sequence of events involving ER stress and Ca^2+^ overload. Our findings provide new insights into the pathogenesis of smoking-affected atherosclerosis and will aid in identifying a novel therapeutic target of RIPK3 to prevent atherosclerosis progression in smoking patients.

### Supplementary Information

**Additional file 1.**

## Data Availability

The datasets generated for this study are available on request to the corresponding author.
