# Supplementary material for: Cigarette tar accelerates atherosclerosis progression via RIPK3-dependent necroptosis mediated by endoplasmic reticulum stress in vascular smooth muscle cells
Source: Cell Commun Signal. 2024 Jan 16;22:41. doi: 10.1186/s12964-024-01480-6 (PMC10790416; doi:10.1186/s12964-024-01480-6)
Supplement: Supplementary file 1 — Additional file 1. [file 12964_2024_1480_MOESM1_ESM.docx]

**
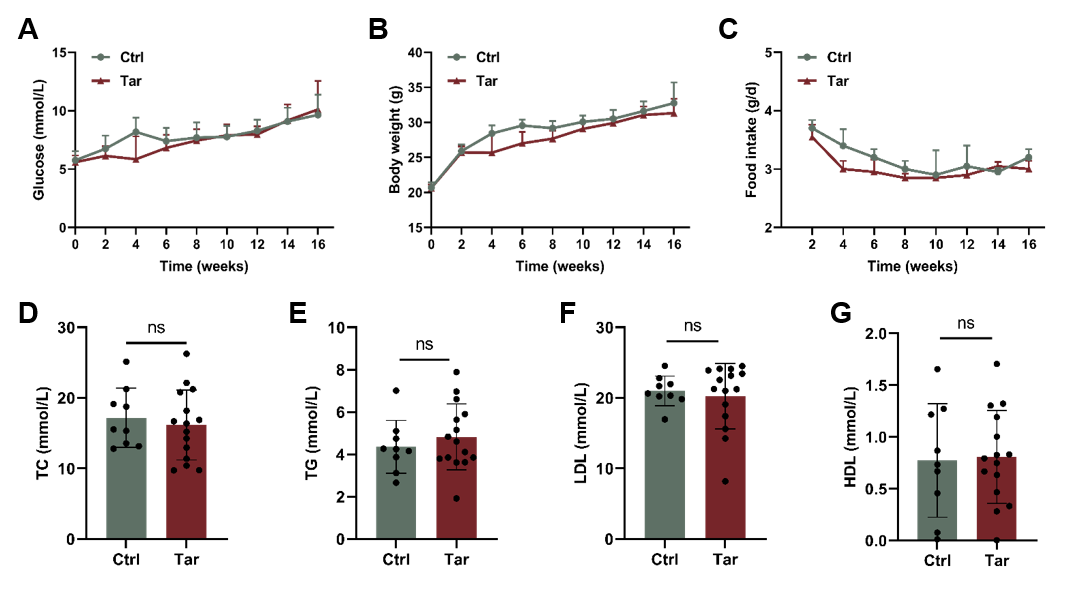
**

**Supplement Figure 1. Baseline characteristics of mice.**

ApoE^-/-^ mice were fed with high-fat diet and injected intraperitoneally with or without cigarette tar (40 mg/kg/day) for 16 weeks. **A.** Blood glucose of mice. **B.** The body weight of mice. **C.** Food intake of mice. **D-E.** Cholesterol and triglycerides levels of mice. **F-G.** LDL and HDL levels of mice. Data were expressed as mean ± SD. *^ns^p >0.05*.


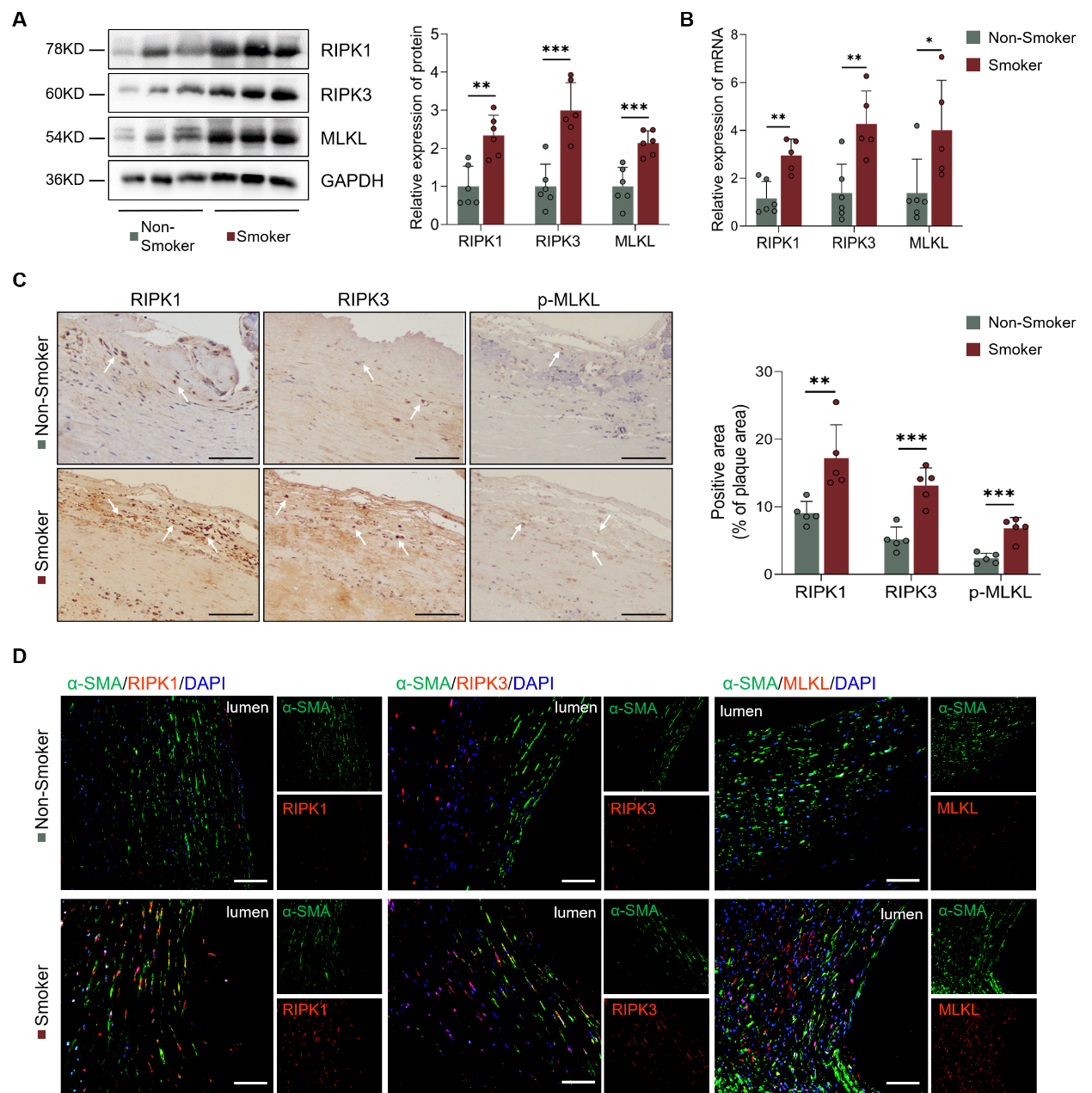


**Supplement Figure 2. Necroptosis is activated in the atherosclerotic plaque of smokers.**

**A.** Representative immunoblots of necroptotic proteins (RIPK1, RIPK3, MLKL) and quantified protein levels in carotid plaques from smokers and non-smokers with atherosclerosis. n=6. **B.** The mRNA expression levels of RIPK1, RIPK3, and MLKL in carotid plaques with atherosclerosis. n=5-6. **C.** Representative images and quantification of RIPK1, RIPK3, and p-MLKL staining on human carotid plaques with atherosclerosis. Scale bar: 100 μm. n=5. **D.** Immunofluorescent staining to detect the co-localization of α-SMA and RIPK1, RIPK3, MLKL in human carotid plaques. Scale bar: 100 μm. n=5. Data were expressed as mean ± SD. **p < 0.05, **p < 0.01, ***p < 0.001.*


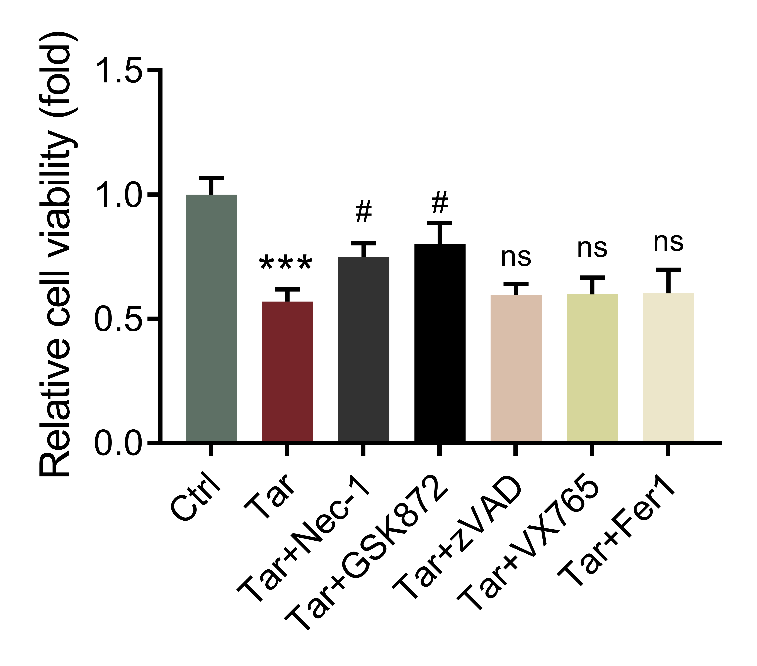


**Supplement Figure 3. Nec-1 or GSK872 reversed the cell viability decreased by tar.**

VSMCs were pretreated with Nec-1 (10 μM), GSK872 (10 μM), zVAD (10 μM), VX765 (10 μM), and Fer1 (5 μM) respectively, then treated with tar (100 μg/ml) for 12 hr. The graph showed the relative cell viability detected by the CCK8 kit. n=3. Data were expressed as mean ± SD. ****p < 0.001*, compared with the Ctrl group; *^#^p < 0.05, ^ns^p > 0.05*, compared with the Tar group.


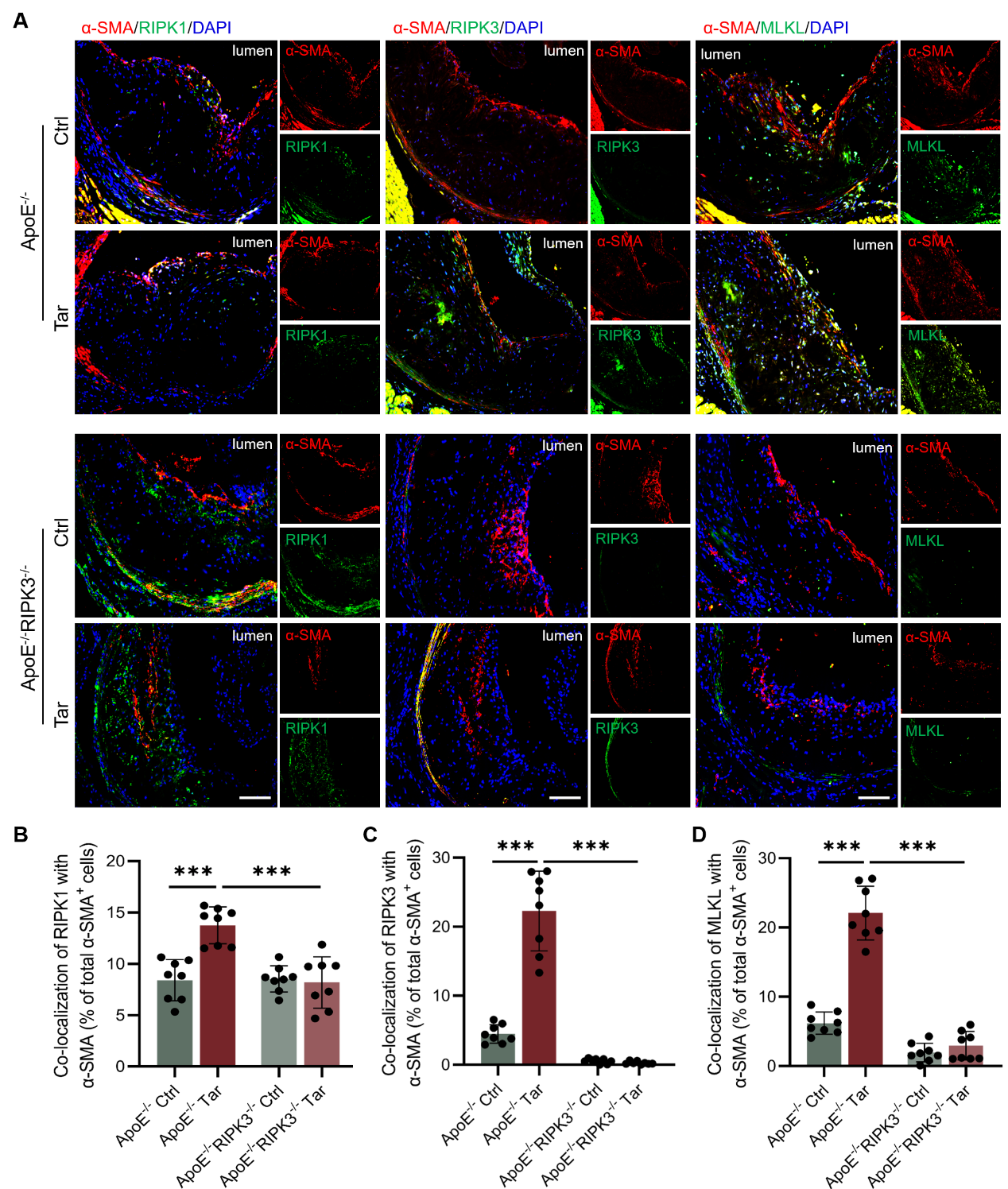


**Supplement Figure 4. RIPK3 deletion decreased the expression of RIPK1 and MLKL in VSMCs.**

**A-D.** Immunofluorescent staining images and quantification of the co-localization of α-SMA and RIPK1, RIPK3, MLKL in aortic roots of mice. Scale bar: 100 μm. n=8. Data were expressed as mean ± SD. ****p < 0.001*.
